# Vaccination in Elite Athletes

**DOI:** 10.1007/s40279-014-0217-3

**Published:** 2014-07-02

**Authors:** Barbara C. Gärtner, Tim Meyer

**Affiliations:** 1 Institute for Microbiology and Hygiene, Saarland University, Faculty of Medicine and Medical Center, Building 43, 66421 Homburg/Saar, Germany; 2 Institute of Sports and Preventive Medicine, Saarland University, Saarbrücken, Germany

## Abstract

Public health vaccination guidelines cannot be easily transferred to elite athletes. An enhanced benefit from preventing even mild diseases is obvious but stronger interference from otherwise minor side effects has to be considered as well. Thus, special vaccination guidelines for adult elite athletes are required. In most of them, protection should be strived for against tetanus, diphtheria, pertussis, influenza, hepatitis A, hepatitis B, measles, mumps and varicella. When living or traveling to endemic areas, the athletes should be immune against tick-borne encephalitis, yellow fever, Japanese encephalitis, poliomyelitis, typhoid fever, and meningococcal disease. Vaccination against pneumococci and *Haemophilus influenzae* type b is only relevant in athletes with certain underlying disorders. Rubella and papillomavirus vaccination might be considered after an individual risk–benefit analysis. Other vaccinations such as cholera, rabies, herpes zoster, and Bacille Calmette–Guérin (BCG) cannot be universally recommended for athletes at present. Only for a very few diseases, a determination of antibody titers is reasonable to avoid unnecessary vaccinations or to control efficacy of an individual’s vaccination (especially for measles, mumps, rubella, varicella, hepatitis B and, partly, hepatitis A). Vaccinations should be scheduled in a way that possible side effects are least likely to occur in periods of competition. Typically, vaccinations are well tolerated by elite athletes, and resulting antibody titers are not different from the general population. Side effects might be reduced by an optimal selection of vaccines and an appropriate technique of administration. Very few discipline-specific considerations apply to an athlete’s vaccination schedule mainly from the competition and training pattern as well as from the typical geographical distribution of competitive sites.

## Key Points

**Table:** 

Risk–benefit analysis of vaccination in elite athletes differs significantly from that of the general population, providing the rationale for specific vaccination guidelines
Risk of infection is higher in athletes due to worldwide traveling and close contact with teammates or opponents. Moreover, consequences of infection are more serious, since even mild infections might be relevant for individual performance
Adverse reactions could be reduced by selecting the optimal vaccine, the optimal time point for vaccination and the correct vaccination technique

## Introduction

Prevention of infection is a key issue in the healthcare of athletes. Exposure prophylaxis (e.g. avoiding mosquito or animal bites, avoiding contact with infected individuals, food, and personal hygiene) and vaccination play major roles in these matters. Although this article focuses on vaccination of adult athletes only, vaccination of the staff or family members is similarly important to create herd immunity and to reduce the risk for the athlete to get in contact with an infectious agent.

Among team doctors and other physicians there exists some uncertainty about the most appropriate vaccination regimens in athletes. Some typical circumstances of athletes’ daily life, such as frequent travelling to foreign countries or close contact with teammates and opponents, might indicate the need for a modification of recommended vaccination schedules. In addition, intense physical activity of training and competition with its possible effects on the immune function can affect decisions about execution and timing of vaccination. Such a complex situation warrants a detailed review of the most current scientific literature with regard to these issues. It is intended to deduct valid recommendations for the available vaccines from an international perspective. An important prerequisite for an immunization campaign in athletes is probably the acceptance of vaccination requirements by opinion formers in the clubs or organizations. Moreover, all staff members should be vaccinated as role models and to provide herd immunity.

## Principles of Vaccination in Elite Athletes

### Existing Vaccination Recommendations

In many countries, considerably different vaccination guidelines have been established and change over time [1–5]. These guidelines target mainly on public health issues and focus on the general population rather than on individuals with a different benefit–risk profile. Several potential reasons exist for not recommending an available vaccine for the general population or for a defined subgroup. Besides few vaccines with an adverse medical risk–benefit ratio, the majority of vaccines are not generally recommended since the medical benefit is not regarded sufficiently balanced with the costs from the view of the general population (cost effectiveness), although being potentially beneficial in a specific individual [6, 7]. Some guidelines address this problem by an ‘opening clause’ indicating that even vaccinations not recommended by the guideline may be beneficial on an individual basis. Thus, they might be offered despite the lacking general recommendation [2].

### Risk–Benefit Balance for Vaccination in Elite Athletes

In many aspects, the medical risk–benefit balance in elite athletes differs significantly from that of the general population [8]. Obviously, this might also affect cost-effectiveness considerations.

#### Vaccination is More Beneficial

Infections have a different significance in competitive sports. For elite athletes, even mild diseases that would never cause absenteeism in the general population are relevant for their individual performance. Seemingly trivial infections might well impair general well-being (or the athlete’s perception of being perfectly prepared) and represent an obstacle for the realization of maximal performance. Also, with the knowledge of a player’s infection, team coaches may tend to leave them on the bench. The same is true for long-lasting infections and post-infectious periods without full recovery of physical performance. When white-collar workers have already gone back to work, elite athletes are still clearly impaired or even unable to train and compete. Furthermore, some infections which typically cause only mild diseases in rare cases might result in severe complications such as myocarditis. This is a well known fact for at least the influenza virus. Athletes are potentially more prone to such organ infections than sedentary individuals, particularly during strenuous training and competition. Although evidence is mainly from animal studies, the severity of the disease renders this assumption tenable [9–11].

The spectrum of infectious agents potentially affecting athletes is different from that of the general population. Elite athletes are often frequent travelers and, thus, prone to acquire infections not prevalent in their home countries. Also, they frequently have contact with teammates or opponents from countries with a different profile of endemic diseases. Thus, a worldwide spectrum of infectious agents has to be considered.

Close contact with opponents and teammates favors transmission of many diseases, particularly respiratory-transmitted diseases [12, 13]. Typically, a contact of less than 1–2 m distance is necessary to transmit diseases such as influenza or other respiratory-transmissible infectious agents such as varicella [14, 15]. For blood-borne diseases, the transmission risk due to sport seems to be less pronounced, however still slightly higher than in the general population [16, 17]. Even healthy non-immune athletes being exposed to an infectious agent (contact with a diseased individual) might be excluded from training and competition for medical reasons. Usually such exclusion has to last for the complete incubation period of a disease (up to 3 weeks). That does not apply to vaccinated and thus immune athletes. Such a kind of prophylaxis was performed during the H1N1 influenza pandemic or recently during a mumps outbreak in the French rugby league [18].

Taken together, these facts argue in favor of a more aggressive vaccination policy since the elite athletes might benefit from a vaccine far more than the general population.

#### The Risk from Vaccination is Higher

Similar to the risk due to infection, the risks of vaccination are aggravated in athletes. At present, no long-term adverse effects occurring some years after vaccination have been identified using post-licensure passive surveillance notification systems such as the vaccine adverse event reporting system (VAERS; https://vaers.hhs.gov/index) in the US or similar notification systems [19]. Thus, only side effects manifesting shortly after vaccination have to be considered. These side effects of vaccines include (1) local reactions at the site of inoculation with the vaccine; (2) generalized reactions, e.g. allergic reaction or a usually mild disease, including fever, lymph node swelling, and headache; and (3) vaccine-specific symptoms that might mimic the disease aimed to prevent when using live vaccines.

##### Local Reactions

Local reactions occur frequently and early after application (6–72 h) and resolve within not more than 7 days [20, 21]. These local reactions are of minor importance in the general population and typically do not interfere relevantly with business requirements [22–25]. However, this is not necessarily true in athletes. Modern vaccines can be administrated by injection (intramuscular, subcutaneous, and intradermal), as a nasal spray or as an oral vaccine (Table 1). Local reactions differ clearly, dependent on the route of administration. In vaccines administered by injection, pain, swellings, or indurations are frequently found. In a few cases (~1 % in children) itchy subcutaneous nodules (granuloma) appear. Aluminium-adsorbed vaccines administrated to the subcutis cause this phenomenon, which is suspected to be related to a contact allergy to aluminium [26].

**Table 1:** Administration of vaccines

Vaccine	Route of administration				
	Intramuscular	Subcutaneous	Intradermal	Oral	Intranasal
Measles	X^a^	X			
Mumps	X^a^	X			
Rubella	X^a^	X			
Varicella		X			
Yellow fever	X	X			
Herpes zoster		X			
Cholera				X	
Pertussis	X				
Tetanus/diphtheria	X				
Tick-borne encephalitis	X				
Influenza	X	X	X		X
Hepatitis A	X	X^b^			
Hepatitis B	X	X^b^			
Poliomyelitis	X	X^b^		X	
Pneumococcal disease	X^c^	X^d^			
Meningococcal disease	X^c^				
Typhoid fever	X	X		X	
Japanese encephalitis	X	X^b^			
Rabies	X				
Papillomavirus	X				
Bacille Calmette–Guérin (BCG)			X		

^a^In combination with varicella vaccine, only a subcutaneous injection is possible

^b^Intramuscular injection preferred; only when an intramuscular injection is not possible, a subcutaneous injection should be considered

^c^Conjugate vaccines should only be administrated intramuscularly

^d^Polysaccharide vaccine might be administered intramuscularly or subcutaneously

When using the intradermal route (e.g. for influenza), local reactions are mainly erythema (7 % of all vaccinations) and swelling (15–30 %) [27, 28]. Vaccines applied intranasally result in a significantly higher rate of local symptoms, as shown for the influenza vaccine. A runny nose was reported in ~50 % and a sore throat in ~25 % [29]. Very few vaccines are administrated orally and replicate in the gut. This replication might result in gastrointestinal symptoms and vaccinees can excrete the vaccine virus or bacteria for some weeks or months [30–32].

##### Generalized Reactions

Next to syncopes or collapses, which are more related to the injection itself than to the vaccine (see Sect. 5), generalized reactions may occur, including fever, headache, fatigue, or lymph node swelling. Depending on the definition of adverse reactions, the vaccine and the vaccinated cohort, the frequency of generalized adverse reactions might differ significantly [33–35]. These generalized reactions indicate an immunological reaction caused by the vaccine [36–38]. These reactions might be present during the first days after vaccination [35, 39, 40]. Next to these usually mild general reactions, severe reactions rarely occur, such as anaphylactic or anaphylactoid reactions, and they probably have the same relevance for sportsmen and the general population.

Severe acute allergic reactions rarely occur (~1:10 million doses for influenza vaccine or measles vaccine) and manifest immediately after vaccination (seconds to 1 h) [41, 42]. However, anaphylactoid reaction is more common (~1:100,000) [42]. Subacute allergic reactions appear a little later (some hours–2 days) and are usually characterized by urticaria, swellings, and exanthema. Delayed allergic reactions manifest some days to 1 week after vaccination (e.g. vasculitis after hepatitis B vaccination [43].

Guillain–Barré syndrome (GBS) is a very rare event after vaccination with modern vaccines and occurs with a frequency of 1:1 million vaccinations or less [44, 45]. Other hypothetical side effects of vaccination have almost been ruled out, such as multiple sclerosis, diabetes mellitus type 1, or autism [46–49].

##### Vaccine-Specific Reaction

Live vaccines against measles, mumps, rubella, varicella, yellow fever, cholera, poliomyelitis, or typhoid fever might cause a mild vaccine disease [50, 51]. This is due to the fact that live vaccines are only attenuated and viruses or bacteria replicate in the body. Thus, a mild disease might occur, mimicking the disease the vaccine was designed for. Fever and/or a few vesicles after varicella vaccination, elevated transaminases after yellow fever vaccination, meningitis after mumps vaccination, benign thrombocytopenic purpura after measles vaccination, or arthritis after rubella vaccination have been reported [48]. These symptoms normally occur after 10–14 days at the peak of replication. This should be considered for the timing of a vaccination (see Sect. 4). The frequency of some of these reactions is related to the vaccine strain, as shown for aseptic meningitis after mumps vaccination. Strains used in older vaccines such as Urabe had a much higher rate of aseptic meningitis compared with modern strains such as Jeryl Lynn strain [47, 52, 53].

### Rationale for Vaccination Guidelines of Elite Athletes

As a result of these considerations, elite athletes need special vaccination guidelines that differ from the ones for the general population. Taken together, the benefits from vaccination and the risk from side effects have to be thoroughly balanced for the situation of an individual athlete. Therefore, we discuss the use of vaccines in adult elite athletes, excluding anthrax and smallpox vaccine, which are provided for military service only in a few countries, and excluding rotavirus vaccine since this vaccine is only licensed for infants.

## Indications for Vaccination in Elite Athletes

### Vaccines Recommended for All Athletes

For adult elite athletes, the inactivated vaccines against tetanus, diphtheria, pertussis, influenza, hepatitis A, hepatitis B, and the live vaccines against measles, mumps and varicella (if immunity is not proven by a natural infection) are uniformly recommended.

#### Tetanus and Diphtheria

Tetanus and diphtheria vaccines are implemented in almost all national guidelines and usually athletes have been vaccinated during early childhood with basic immunization (TD). However, in adults, a 10-yearly booster dose with a reduced diphtheria component (Td) is recommended. It might be worth mentioning that in many sports, bodily contact with soil and dust cannot be avoided, as well as the occurrence of wounds, both of which might favor the acquisition of *Clostridium tetani*. Although the risk of acquiring diphtheria is low, both infections are very severe and often associated with serious complications, which further justify their prevention by well-established vaccinations.

#### Pertussis

Pertussis vaccination for adults is only recommended by a few national guidelines in adults such as in Germany, Italy, France, UK, Austria, and the US, whereas it is not recommended for all adults in most other EU countries, Russia, Brazil, or Australia. However, there is growing evidence that pertussis is affecting adults, resulting in a variety of severe symptoms of the respiratory system that might last for many weeks and months. During the last years, the median age of infected individuals increased in many countries and thereby adults came into the focus of vaccination [54]. The risk of a clinically relevant disease is around 1:500 per year and vaccination reduces this risk by over 90 % at least for the first 2–3 years after vaccination [55]. The only licensed vaccine for adults is an acellular vaccine (with less side effects compared with a whole-cell vaccine) with a reduced antigen content compared with childhood vaccines used for basic immunization. At present, vaccination against pertussis in adults is only feasible using a combined vaccine together with tetanus and diphtheria booster dose [56]. It can well happen that pertussis vaccine is indicated but tetanus/diphtheria booster doses were already given within the last years. Earlier, it was suspected that side effects increase when shortening the interval between tetanus–diphtheria pre-vaccination and tetanus–diphtheria–pertussis booster [57]. However, two recently published reports do not support this hypothesis. Even within an interval of only 1 month, adverse reactions did not occur to a higher frequency in individuals recently pre-vaccinated compared with controls [58, 59]. Thus, pertussis vaccination is recommended in athletes because the likelihood of acquiring a severe, long-lasting infection that interferes with training and competition is relevant, and the vaccine-associated side effects seem tolerable.

#### Influenza

Influenza is an important health issue, even in young, healthy adults. The disease might be severe and the virus is highly contagious. This alone might constitute sufficient justification for a recommendation to vaccinate athletes. Such a consideration is based on the fact that even a moderate or mild influenza might cause absence from training and competition for weeks and possibly the loss of a whole season. Unfortunately, the vaccine efficacy differs from season to season and is generally less than that of other vaccines [60, 61]. Influenza vaccination is complicated by the fact that a wide variety of vaccines is available. Next to a live vaccine that is applied intranasally, different inactivated vaccines applied via the intradermal or intramuscular route are available. Moreover, the vaccines differ in the adjuvants used (with and without MF59), influencing the antibody production and the likelihood of adverse reactions. In addition, the antigens in the vaccines are manufactured differently. At present, sub-unit, split, and whole-virus vaccines are available. In the majority of vaccines, the antigens are produced in eggs and less frequently in cell cultures with a slightly different profile in antibody production and side effects. Finally, for the first time this season, some vaccines do not only include the two influenza A (H1N1 and H3N2) and one influenza B components originating from the Victoria or Yamagata lineage as an alternative (trivalent vaccine) but also both influenza B lineages (quadrivalent vaccine). The second influenza B component was implemented since two influenza B lineages are cocirculating without relevant cross protection between each other, resulting in an important lack of protection for the trivalent vaccine. However, the fourth component is included only in a few vaccines commercially available [62–65].

Having this high number of different vaccines in mind when selecting an appropriate vaccine for healthy young adults that should be accompanied by a minimum of adverse effects, the use of adjuvanted vaccines is discouraged (more side effects with a benefit that is mainly detectable in immunosuppressed patients and elderly but less in healthy young adults) [66, 67]. The use of the quadrivalent influenza vaccine seems to be beneficial since quite a high number of influenza virus infections were caused by an influenza B type not included in the seasonal vaccine by the World Health Organization (WHO) recommendation during the last 10 years [68].

Concerning the other vaccine properties, the decision is less clear. An application by intranasal, intradermal, or intramuscular route is accompanied by different profiles of side effects. Efficacy is only slightly different between the intradermal and the intramuscular route. The intradermal application differs from the intramuscular application in the profile of local reactions. Rates of local adverse events were consistently higher after intradermal application, particularly erythema, swelling, induration, and pruritus. However, individuals reported less pain in the muscle after intradermal application [69]. Taking this into account, the optimal administration route varies between athletic disciplines. For a runner, the intradermal route or the deltoid seems preferable, whereas an archer may benefit from an intragluteal injection. The intranasally applied live vaccine (not available with inactivated vaccines) leads to a much higher protection in young children. With increasing age, this effect decreases to a level not different or even lower than for intramuscularly administered vaccines [60, 70]. The live vaccine has some other characteristics that apply to this kind of vaccine only. While replicating in the upper respiratory tract, it is possible that the virus might be transmitted to others within the first 2–3 days (up to 10 days). However, the rate of such transmission seems to be small (<2 %) [71]. The major benefit of this vaccine is its favorable profile of side effects without the typical symptoms of pain, swelling, or induration at the site of vaccination but with a runny nose or nasal congestion. At present, for athletes an intramuscular or intradermal application should be preferred since the live vaccine has not proven its effectiveness in healthy adults sufficiently compared with intramuscular vaccines. The live vaccine seems to be an option only in a few cases with an anaphylactic reaction after intramuscular vaccination or when local reactions at the injection site must be absolutely avoided for sport-specific or other reasons.

It should be kept in mind that in the two hemispheres different vaccines might be recommended and that the influenza season differs considerably due to the climate. This means that influenza can be a risk year-round, and even outside of the typical influenza season when travelling to countries with differing influenza seasons. Especially when travelling to the other hemisphere, a twice-yearly vaccination is essential for optimal protection.

Taken together, vaccination with a quadrivalent intramuscular or intradermal administrated influenza vaccine seems to be the best option for the majority of elite athletes.

#### Hepatitis A

Hepatitis A is frequently found in many countries around the world and is mainly a food-borne disease that is difficult to prevent using simple measures [72, 73]. Its prevalence is higher in countries with moderate climate and poor hygiene levels which are frequently chosen for training camps [74]. Due to the worldwide food market, even countries with typically low endemicity, such as Northern Europe, can be affected nowadays [73]. Thus, it is almost impossible to prevent hepatitis A virus infection by exposure prophylaxis alone. A vaccination is recommended because this disease typically leads to some months of reduced physical performance, and hepatitis A can be easily transferred to teammates and opponents [75].

#### Hepatitis B

Hepatitis B is mainly transmitted by blood or genital secretions. Viral load in infected individuals is rather high, enabling transmission even when only small amounts of infected fluids are transmitted. Thus, small injuries with blood contact to others might be sufficient to transmit the virus [16, 17]. Consequently, the vaccination is relevant in all sports with possible contact to blood and body fluids, such as football, boxing, and hockey, but less so in sports such as tennis or most winter sports [76]. Moreover, hepatitis B is highly prevalent in Africa, parts of Asia, and Latin America. Contact with the healthcare system in these countries may harbor an additional risk. Different hepatitis B vaccines are on the market. Vaccines with various hepatitis B surface antigen (HbsAg) concentrations (10, 20, and 40 µg) are available. Also, the antigens might be different since one vaccine includes not only the small HbsAg but all three subtypes of HbsAg (small, middle, and large) with the benefit of a better immune reaction and similar side effects [77]. In addition, a vaccine with a special adjuvant (AS04) is available that was mainly designed for immunosuppressed individuals [78]. For healthy adults, a 20 µg dose without adjuvant AS04 seems to be sufficient. The other formulations are an option for non-responders with the need for protection [79]. Hepatitis B vaccination is strongly recommended in athletes because of the disease severity (typically several months of no or reduced training and competition eligibility complicated by irreversible organ damage) and its contagiosity (likelihood of transfer to teammates and opponents).

#### Measles, Mumps, and Varicella

**Fig.1:**
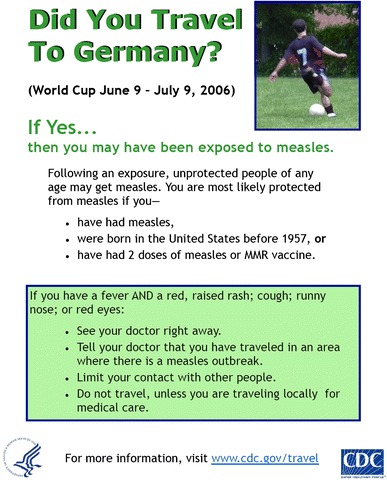
Poster at US airports (this poster was displayed in Boston) after the 2006 World Cup in Germany, since measles occurred during that time (photo reproduced with permission from Prof. Dr. Martina Sester)

Outbreaks with measles have been reported during sport events (as reviewed by Pyne and Gleeson [80]). Measles is an extremely contagious and severe disease with a high rate of complications (pneumonia, otitis, encephalitis) [81, 82]. There is no doubt that elite athletes should be as immune to measles as everyone else. In quite a few countries where big football tournaments took place during the last years, a measles epidemic occurred at the same time (such as 2006 in Germany, 2010 in South Africa, 2008 in Switzerland/Austria, 2012 in Poland/Ukraine; Fig. 1). Measles has an extremely high basic reproduction number *R*_0_ of 7–30 (number of cases one case generates on average over the course of its infectious period, in an otherwise uninfected population) [83]. This means that even a short-lasting contact (e.g. with employees in hotels, shops, contact on streets) might result in an infection.

Mumps infection is a little less severe and contagious compared with measles with an *R*_0_ of 3–10 [84]. However, it causes a general illness in adults with parotitis and often (15–20 %) orchitis and meningitis (10 %) as a complication [85, 86]. Moreover, some sports event had to be cancelled due to mumps [18, 87, 88]. Immunity after vaccination is not as high for mumps as it is for measles, even after two vaccinations. This means that the virus might circulate, even in countries with a high vaccination status [89].

Varicella vaccination is important to prevent chicken pox in all non-immune individuals. The worldwide prevalence of antibodies in young adults is relatively high, mainly due to natural infections. However, up to 10 % of adults are not immune [90]. Varicella virus is highly contagious (*R*_0_ = 7–13) and young adults are often exposed to (their own) children with chicken pox [91].

All these infections (measles, mumps, and varicella) have a more severe course in adults compared with children. This is particularly true for varicella with pneumonia and hemorrhagic varicella often with bacterial superinfection as complications [86, 92, 93]. Thus, there is no doubt that all elite athletes should be immune against varicella.

All three live vaccines should be administrated at least twice in non-immune individuals, with a minimum interval of 4 weeks [94]. It is recommended to use combined vaccines whenever possible [4]. The same applies to rubella vaccination, which is described later (see Sect. 3.2.3).

### Vaccines Recommended in Special Situations

#### Vaccines Recommended due to Epidemiological Reasons Only

##### Tick-Borne Encephalitis, Yellow Fever, Japanese Encephalitis

Since tick-borne encephalitis, yellow fever, and Japanese encephalitis are solely vector-borne diseases, they should only be considered when athletes live or travel to the endemic areas, i.e. Eastern, Central and Northern Europe, Northern China, Mongolia, and the Russian Federation for tick-borne encephalitis; Africa and some tropical parts of South America for yellow fever; and parts of China, the Russian Federation’s south-east, and South and South-East Asia (including India and Nepal) for Japanese encephalitis [95–97]. Consequently, tick-borne encephalitis was recommended before the 2008 European football championship in Switzerland and Austria. When athletes travel to these regions, recommendations do not differ from the general population due to the severity of these diseases.

##### Poliomyelitis

Poliomyelitis is only rarely found worldwide and at present it occurs only in a few countries with major social and political problems, such as Afghanistan, Pakistan, Syria, Somalia, and Nigeria [98]. Without direct contact with these countries (or indirectly through teammates), the risk of acquiring this infection is rather low [98]. An oral live vaccine and an inactivated vaccine for intramuscular injection are available. Again, both have a different profile of adverse events; the gut is more often involved when using the oral vaccine [99], whereas the inactivated vaccine causes side effects at the injection site [5]. Since the live vaccine harbours the rare risk to re-mutate to a pathogen, possibly causing outbreaks, it is discouraged in areas certified by the WHO as poliovirus-free [40, 98]. The WHO updates the list of countries with and without poliomyelitis, as well as the poliomyelitis-free region, on their web page [1]. The vaccine to be chosen for athletes should be the same as recommended by the national guideline for the general population.

##### Typhoid Fever

Typhoid fever is found in some endemic areas, such as several Asian regions of Russia and neighboring countries, and in parts of South and South-East Asia, Africa, and South America [100]. Within the last 10 years, there were large outbreaks in the Democratic Republic of Congo and Haiti [100]. However, the risk of transmitting the bacteria is rather small when taking the travel habits of elite athletes into consideration since these bacteria are mainly transmitted in the setting of poor hygiene. At present, three different vaccines are available: an oral live-attenuated vaccine (Ty21a strain of *Salmonella typhi*), a parenteral inactivated vaccine (Vi polysaccharide vaccine, one dose), and a newly licensed capsular polysaccharide vaccine (Vi-rEPA, two doses) for parenteral use. Efficacy seems to be higher using the new vaccine (<75 % seroconversion) compared with the two others (~50 %) [101, 102]. The oral vaccine rarely has side effects that mainly consist of abdominal discomfort, nausea and vomiting, whereas with the parenteral vaccines the local reactions at the site of injection dominate [103]. Theoretically, the live vaccine’s effect can be diminished by the use of antibiotics. It is thus recommended that this vaccine should be administered not earlier than 24 h after an antimicrobial dose [104].

##### Meningococcal Disease

Meningococcal vaccination is important at least when travelling to countries with high endemicity (sub-Saharan Africa from Senegal to Ethiopia). Moreover, outside of the endemic areas, meningococcal vaccination is relevant since sporadic meningococcal meningitis with complication may develop in healthy individuals, with a high fatality rate of 10–50 %. The disease peaks in children <6 years of age and in adolescents and young adults, and thus might play a role for young athletes [105, 106].

Similar to pneumococcal vaccination (see Sect. 3.2.2), a conjugate and a polysaccharide vaccine are available. Both vaccines cover the same subtypes. Immune response to conjugate vaccines is much better, clearly favoring this type [107, 108]. The vaccines currently available cover the serotypes A, C, W135, and Y [109]. In endemic regions, serotype A is the most prevalent, whereas serotype B dominates in non-endemic regions [110, 111]. Recently, a new vaccine was licensed targeting serotype B. Experience with this vaccine is very limited in healthy adults, thus it appears too early to recommend vaccination in athletes. If vaccination with the new serotype B vaccine is considered, it is strongly recommended to apply it in a resting period since adverse events with myalgia, arthralgia, headache, and fever are frequently found [112, 113].

Taken together, vaccination against meningococcal disease with a conjugate vaccine covering the serotypes A, C, W135, and Y is recommended when travelling to endemic areas [109–111]. Vaccination against serotype B cannot be recommended at present since available data are too limited.

#### Vaccines Recommended due to an Underlying Disorder

##### Pneumococcal Disease

Vaccination against pneumococcal disease is not implemented in national guidelines for young healthy adults but only for the elderly and for patients with certain underlying disorders [2, 4, 114]. For athletes, this vaccination should only be considered in the case of immunocompromizing conditions, functional or anatomic asplenia, cerebrospinal fluid (CSF) leaks, or cochlear implants [114]. Moreover, it is recommended for patients with chronic lung diseases such as asthma [1–5].

Two vaccines are available eliciting a different quality and quantity of immune response: a conjugated vaccine including 13 serotypes (PCV-13) at present and a polysaccharide vaccine with 23 serotypes (PPSV-23). Vaccination schedules are different for vaccine-naïve individuals and individuals prevaccinated with the polysaccharide vaccine. At present, it is considered advantageous for many underlying diseases that individuals receive one dose of the conjugate vaccine. A vaccine-naïve individual should receive the conjugated vaccine first followed by the polysaccharide vaccine after 8 weeks, since the opsonophagocytic activity seems to be reduced when the two vaccines are switched [115]. In a few diseases a booster vaccination is necessary. In these individuals the polysaccharide vaccine should be administered 5 years after the last vaccination. In individuals prevaccinated with the polysaccharide vaccine one dose of the conjugate vaccine should be used ≥1 year after the last dose of the polysaccharide vaccine [114, 116, 117].

##### *Haemophilus influenzae* Type b (Hib)

Similar to the pneumococcal vaccine, use of the Hib vaccine is only advised in the rare event of an asplenic athlete. One dose of Hib vaccine should then be administered [118].

#### Vaccines with a Critical Medical Benefit–Risk Ratio in Athletes

##### Rubella

Rubella infection causes a much milder disease compared with measles or mumps and is mainly asymptomatic, with a rash as the most prominent manifestation that is difficult to distinguish from allergic reaction. Fever and other complications are rare [119, 120]. Public health guidelines focus on the prevention of prenatal rubella infection that may cause embryopathy. Thus, vaccination of males targets to break the chain of infections and to reduce the risk for pregnant women and not to prevent the disease in non-pregnant women. One important complication of both the infection and the vaccination is arthritis for some weeks/months [121–123]. In a randomized controlled trial (strain RA27/3 versus saline), women vaccinated with rubella vaccine reported arthritis in ~30 % (controls ~20 %) [124]. Most of the data on arthritis after vaccination originate from studies of postpartum vaccination and thus only women were included. It seems that the risk for arthritis after vaccination in men is by far lower [122]. Thus, the risks and benefits of a rubella vaccination have to be considered carefully in an athlete since arthritis might be considered a more severe problem than in the general population.

##### Papillomavirus

Papillomavirus vaccination might prevent papillomavirus-associated genitoanal lesions, cancer, and condyloma accuminata. It is unclear if athletes are at a higher risk of acquiring sexually transmitted diseases since data on promiscuity in elite athletes are not available. Vaccination of adults is only recommended in a few countries [125]. Two vaccines are available: a bivalent vaccine including human papillomavirus (HPV) 16 and 18, with an adjuvant AS04 and a quadrivalent vaccine without this adjuvant and, additionally, HPV 6 and HPV11 (to prevent condyloma accuminata) [126]. Since the adjuvanted bivalent vaccine has a higher cross-protection against other high-risk types causing cancer, this vaccine seems to be of advantage for women because in women protection against cancer has the highest significance [127, 128]. In men, prevention of condyloma accuminata seems to be of more concern since HPV-associated cancer in men is found less frequently. Thus, for men, vaccination with the quadrivalent vaccine including HPV6 and 11 is probably the best option [125, 129].

### Vaccines not Relevant to Athletes

#### Cholera

Vaccination against cholera does not seem to be relevant since cholera is a disease of very poor hygienic level, classically confined to refugee camps or slums.

#### Rabies

Rabies vaccination is not recommended since the vaccine has a high number of considerable side effects and the disease might be prevented by exposure prophylaxis. It should be possible to prevent animal bites in athletes by other measures and, when an incident occurs, post-exposure prophylaxis can be administered even after the bite [130].

#### Herpes Zoster

Vaccination against herpes zoster (shingles) does not seem to be indicated since herpes zoster is only very rarely found in athletes. There are some anecdotal reports of zoster occurrence in endurance athletes during highly strenuous training periods but due to their scarcity no conclusion of compromised immunity in this particular athlete population (as a typical precondition for zoster) can be drawn. Also, vaccination could not be recommended at present since there are no data on the efficacy in young adults but only in individuals >50 years of age [51, 131]. The vaccine was introduced only a short time ago and thus it is too early to draw any conclusions on a population for which the vaccine has not been tested so far.

#### Bacille Calmette–Guérin (BCG)

Bacille Calmette–Guérin vaccination is implemented in many countries for childhood vaccination, whereas vaccination in adulthood is usually not recommended. Although tuberculosis might be relevant in athletes, especially originating from countries with high endemicity, the vaccination of adult teammates does not seem to be beneficial [132]. This is due to the fact that the vaccination has a number of severe adverse events since it is a live vaccine and the bacteria replicate in the body, which might cause local infection and spread to the regional lymph nodes, accompanied by lymphadenitis. In rare events, abscesses can occur. Moreover, since the vaccine does not protect from primary tuberculosis, the chance of preventing transmission even within a team is very limited.

## Timing of Vaccinations

Timing of vaccinations should be chosen with the purpose of minimizing interference with training and competition and making sure that the immune reaction is not temporarily impaired. Relevant side effects after inactivated vaccines can be expected within the first 2 days after vaccination, whereas after live attenuated vaccines they are more likely to occur after 10–14 days when the replication of the vaccines in the body peaks (see Sect. 2). Under these constraints, an appropriate time for vaccination which is not acutely indicated would be at the onset of resting periods or shortly prior to the winter and summer breaks.

Although indications for increased frequency of upper respiratory tract infections after strenuous exercise, such as marathon races, exist [133–135], measurable changes in immune cell number and function have mainly been documented within 2 h post-exercise [134, 136, 137] Theoretically, a compromised immune reaction to vaccinations can be derived from such observations. However, it has been shown that influenza vaccination did not lead to decreased titers when conducted immediately after physical activity and that acute exercise even increased antibody responses in pneumococcal vaccination [138–141]. In another study in elite athletes, titers after hepatitis B vaccination were identical to the general population [142]. Thus, when a vaccination has to be carried out within a training and/or competition period (e.g. influenza), there is no major medical problem with vaccinating shortly after a competition to make the period of time to the next competition as long as possible. Acute exercise might even act as a weak adjuvant, increasing antibody responses slightly in some individuals [138, 139, 141, 143]. In contrast, the pain reaction following the vaccination was clearly diminished when vaccinating 6 h after activity compared with vaccination immediately or 24–48 h after activity [143]. This indicates that 6-h post-exercise might represent a preferable point in time.

## Methods to Reduce Side Effects

Pain, headache, and fever as side effects might be reduced by co-administering substances such as paracetamol or ibuprofen, even though antibody titers can be slightly lower under such circumstances [37]. As already outlined in Sect. 3, another option is to choose a vaccine with a low profile of adverse events. Side effects of vaccines with more potent adjuvants, such as MF59, AS03, or AS04, are usually more intense although accompanied by higher antibody titers [66]. Moreover, the profile of local reactions is mainly based on the route of vaccination. Thus, oral, intranasal, intradermal, and intramuscular/subcutaneous routes of administration have a different local reaction profile.

In vaccines administrated by injection (intramuscularly or subcutaneously), the local adverse events might be partly due to the injection techniques. Thus, it is worth adhering to the correct injection technique (as reviewed by Petousis-Harris [144]). Dependent on the injection site, specific impairments may result (e.g. for running, from buttock pain after a gluteal injection). Obviously, it is advisable to use the non-dominant side for injections in unilateral disciplines such as racquet sports. The skin disinfectant must be completely dry before injection. Two separate needles for filling of the syringe and for injection should be used to avoid granuloma in the subcutis due to aluminium-containing vaccines [26]. If a vaccine is allowed to be administrated using the intramuscular or the subcutaneous route, the intramuscular vaccination seems to be beneficial (higher titer, lower risk of granuloma). Injection in the deltoid should be preferred, although other muscles are possible. It is important that the vaccinee is sitting or lying and the muscle is completely relaxed. Longer needles (25 mm) and a fast speed of injection and withdrawal of the needle (1–2 s) was associated with less pain [145]. An angle of injection of 90° also reduced pain in intramuscular injections.

Other adverse reactions often occurring in adolescents and young adults are syncopes [146, 147]. According to the VAERS, this phenomenon was observed to increase when introducing papillomavirus vaccine, meningococcal B vaccine, and pertussis vaccine. Syncopes or collapses may be found at a frequency of around 1 % [19, 41]. Importantly, not only the syncope itself but secondary injuries such as skull fracture and cerebral hemorrhage are of major concern. In the VAERS reports, around 10 % of all syncopes resulted in hospitalization due to secondary injuries. The majority of syncopes (80 %) occurred within 15 min of vaccine administration, strongly favoring a 15–30 min observation of a vaccinee [147]. This observation might be of particular importance in endurance athletes because there are indications that, in these athletes, vasovagally-induced syncopes are more frequent [148]. The consequence would be a prolonged interval of medical monitoring in vaccinated (endurance) athletes.

## Indications for Titer Control

Since athletes suffer more from side effects of vaccines (as outlined in Sect. 2), unnecessary vaccinations should be avoided. This is possible in individuals with pre-existing immunity due to a natural infection or a previous sufficient vaccination. Titer controls are generally not supported by national guidelines since they are often more expensive than vaccination. Moreover, antibody assays are not standardized, with the risk of highly different and misleading results between the assays [149, 150]. However, they might be well justified in top-level sportsmen to avoid adverse reactions due to an unnecessary vaccination. This is particularly true for live attenuated vaccines being more prone to side effects as well as for athletes from countries where the likelihood of acquiring natural immunity is high, e.g. against hepatitis A or B.

Usually, a documented vaccination by a valid vaccination certificate equals immunity in most cases. In contrast to this rule, a documented vaccination does not necessarily mean that vaccination was performed lege artis. This is why, in certain athletes who have been vaccinated in countries with doubtful (less immunogene) vaccine quality, a titer control might be worthwhile, even in cases with appropriate documentation. Documented examples for less active vaccines are regions in Eastern Europe or Asia; however, this might also apply for other regions [151–155]. When in doubt, vaccination documentation from such countries should not be regarded reliable. After inclusion of a new team member, the vaccination record should be carefully checked and, in case of any doubt, a titer control can be added. This is particularly true for all team members born and raised in countries with a different vaccination schedule. In very important vaccination situations, e.g. with a high risk of infection or in severe diseases, titer control after vaccination might be justified to be able to revaccinate quickly in cases of non-response (Table 2).

**Table 2:** Available vaccines: options for antibody titer controls, risk assessment for athletes, and vaccination schedules

Vaccine	Titer control^a^	Risk assessment for athletes	Vaccination schedule and vaccine
Vaccines recommended for all athletes (see Sect. 3.1)			
Tetanus/diphtheria (Td)	Unnecessary	Tetanus: high risk of skin-penetrating injury in sport. Diphtheria: severe disease	Basic immunization (often in childhood) with at least three shots. Combination with aP reasonable; booster after 10 years
Pertussis (aP)	Unnecessary	Severe disease with relevant impairment of physical capability; frequently of long duration	Combination with Td. Interval between Td and TdaP at least 1 month; avoid proximity to competition (local reactions); combination with poliomyelitis vaccine; no booster
Influenza	Unnecessary	High risk due to epidemic spread, highly contagious	Yearly vaccination. Different seasons and vaccines worldwide. Quadrivalent vaccine recommended
Hepatitis A (HAV)	Only prior to vaccination	High risk during training and competition in risk areas; also possible in first-class hotels	Basic immunization with at least two shots (months 0, 6–12) as single vaccine or in combination with HBV (see HBV); no booster
Hepatitis B (HBV)	Prior to vaccination and 4–6 weeks after the third shot	High risk in cases of contact (sexually, body fluids) with athletes from Africa, Asia, South America, Eastern Europe, or when utilizing the healthcare system in such countries; small risk from possible blood contact during training/competition	Three shots (months 0, 1, 6); shortened schedule available (days 0, 7, 21, 365). When indicated, combination with HAV preferred; booster dose (only HBV) after 10 years. In low-responders (anti-Hbs 10–100 IU/L) single re-vaccination without further titer control; in non-responders (anti-Hbs-titers <10 IU/L) up to three re-vaccinations, vaccines with high antigen content preferred
Measles (M)	Yes	Severe disease with complication in adulthood, highly contagious, frequent small-area epidemics	Two shots with a min. interval of 4 weeks; combined vaccine preferred [MM(R)V]; no vaccination when immunity is proven by titer control; no additional vaccination after two shots without titer
Mumps (M)			
Varicella (V)			
Vaccines recommended due to epidemiological reasons only (see Sect. 3.2.1)			
Tick-borne encephalitis	Unnecessary	High infection risk during outdoor activities; increasing pathogenicity with increasing age. Eastern, Central and Northern Europe, Northern China, Mongolia, and the Russian Federation	Basic immunization with at least three shots (months 0, 1–3, 9–12); shortened schedule possible (days 0, 7, 21 + 12–18 months); booster dose 3–5 years, pay attention to manufacturer’s advice
Yellow fever	NA	Severe disease, widely spread in Africa and South America. International travel regulations	Single vaccination, protection is assumed to remain lifelong
Japanese encephalitis	NA	Risk only during stays of longer duration (several months, years) in rural areas of Asia (parts of China, the Russian Federation’s south-east, and South and South-East Asia)	Two shots with a min. interval of 4 weeks; booster after 1 year
Poliomyelitis (P)	NA	Very small risk in only a few areas. Risk with close contact to population. Currently re-occurrence in countries where the disease had been eliminated years ago	Basic immunization (typically in childhood) with at least two to four shots depending on the vaccine. In adults without immunity, the complete schedule should be administered. When travelling into endemic areas, a single booster dose is recommended, possibly as combined vaccine TdaPP
Typhoid fever	NA	Low transmission risk, typically bound to low hygiene and contact to local population (Asia, Africa, South America)	Inactivated vaccines (single shot) or live oral (day 0, 3, 5) preferred, heat-inactivated vaccine in combination with HAV possible; booster after 3 years with heat-inactivated vaccine or yearly with live oral vaccine
Meningococcal disease	NA	Low transmission risk but severe disease; important when travelling to the “meningitis belt” (Northern Africa, Arabic countries)	Single vaccination with conjugate vaccine against four types (A, C, W135, Y) preferred; no booster
Vaccines recommended due to an underlying disorder (see Sect. 3.2.2)			
Pneumococcal disease	NA	Low transmission risk but severe disease in patients with underlying disorders	For optimal protection, start with 13-valent conjugate vaccine (PCV-13) and, 8 weeks later, 23-valent polysaccharide vaccine (PPSV-23). If patient already had PPSV-23, the PCV-13 should be added after 1 year. In rare cases, booster after 5 years
Hib	NA	Risk only in patients with underlying disorders	Single dose, no booster
Vaccines with a critical medical benefit–risk ratio in athletes (see Sect. 3.2.3)			
Rubella (R)	Yes	Typically mild disease; frequent complication in adults: arthritis	See measles
Papillomavirus (HPV)	NA	Identical risk to general population	Three shots (0, 1–2 m, 6 m). Special adjuvanted vaccine (AS04; HPV 16 and 18) with higher titers and more local reactions compared with quadrivalent vaccine (HPV 16, 18, 6, 11)
Vaccines not relevant in athletes (see Sect. 3.3)			
Cholera	NA	Indication only in cases of competition or training camps in endemic areas (extremely rare)	Oral vaccination; two doses (day 0, 7), booster after 2 years
Rabies	NA	Low risk of transmission, high risk of side effects; exposure prophylaxis possible; post-exposure vaccination effective	Post-exposure prophylaxis: vaccine (day 0, 3, 7, 14, 28, possibly 90) and hyperimmunoglobulin (single application)
Herpes zoster	NA	Very rare in athletes	Single vaccination, currently only recommended for adults >50 years of age
BCG	NA	Low protection and relevant adverse events	Single intradermal application

*Hib* *Haemophilus influenzae* B, *BCG* Bacille Calmette–Guérin, *Anti-Hbs* antibodies against hepatitis B surface antigen, *min*. minimal, *NA* antibody titer controls are not available, or assays are not implemented in routine diagnostics but only in research laboratories

^a^Titer controls prior to vaccination aimed to avoid vaccination in case of a positive titer; titer control post-vaccination aimed to detect non-responders qualifying for a re-vaccination

## Conclusions

The special situation of elite athletes justifies specified vaccination guidelines that partly differ from public health guidelines. The risk of side effects could be reduced by a correct vaccine and vaccination technique and by the timing of vaccination. All staff members should also be vaccinated to increase the acceptance of vaccination by the athlete.
